# Resource use and efficiency, and stomatal responses to environmental drivers of oak and pine species in an Atlantic Coastal Plain forest

**DOI:** 10.3389/fpls.2015.00297

**Published:** 2015-05-07

**Authors:** Heidi J. Renninger, Nicholas J. Carlo, Kenneth L. Clark, Karina V. R. Schäfer

**Affiliations:** ^1^Department of Biological Sciences, Rutgers, The State University of New JerseyNewark, NJ, USA; ^2^Department of Earth and Environmental Sciences, Rutgers, The State University of New JerseyNewark, NJ, USA; ^3^Silas Little Experimental Forest, Northern Research Station, United States Department of Agriculture Forest ServiceNew Lisbon, NJ, USA

**Keywords:** photosynthesis, canopy conductance, sap flow, nitrogen-use efficiency, water-use efficiency

## Abstract

Pine-oak ecosystems are globally distributed even though differences in anatomy and leaf habit between many co-occurring oaks and pines suggest different strategies for resource use, efficiency and stomatal behavior. The New Jersey Pinelands contain sandy soils with low water- and nutrient-holding capacity providing an opportunity to examine trade-offs in resource uptake and efficiency. Therefore, we compared resource use in terms of transpiration rates and leaf nitrogen content and resource-use efficiency including water-use efficiency (WUE) via gas exchange and leaf carbon isotopes and photosynthetic nitrogen-use efficiency (PNUE) between oaks (*Quercus alba*, *Q. prinus*, *Q. velutina*) and pines (*Pinus rigida*, *P. echinata*). We also determined environmental drivers [vapor pressure deficit (VPD), soil moisture, solar radiation] of canopy stomatal conductance (G_S_) estimated via sap flow and stomatal sensitivity to light and soil moisture. Net assimilation rates were similar between genera, but oak leaves used about 10% more water and pine foliage contained about 20% more N per unit leaf area. Therefore, oaks exhibited greater PNUE while pines had higher WUE based on gas exchange, although WUE from carbon isotopes was not significantly different. For the environmental drivers of G_S_, oaks had about 10% lower stomatal sensitivity to VPD normalized by reference stomatal conductance compared with pines. Pines exhibited a significant positive relationship between shallow soil moisture and G_S_, but only G_S_ in *Q. velutina* was positively related to soil moisture. In contrast, stomatal sensitivity to VPD was significantly related to solar radiation in all oak species but only pines at one site. Therefore, oaks rely more heavily on groundwater resources but have lower WUE, while pines have larger leaf areas and nitrogen acquisition but lower PNUE demonstrating a trade-off between using water and nitrogen efficiently in a resource-limited ecosystem.

## Introduction

Forest ecosystems dominated by pines and oaks are distributed globally (Richardson and Rundel, 1998; Nixon, 2006) including locations in the Eastern United States (Nowacki and Abrams, 2008), Southwestern North and Central America (Kolb and Stone, 2000; Gómez-Mendoza and Arriaga, 2007), the Mediterranean (Guehl et al., 1995; Díaz, 2006), and Alpine Europe (Weber et al., 2007; Zweifel et al., 2007; Sterck et al., 2008; Eilmann et al., 2009). Pine-oak forests tend to occur in water-limited systems where either strong seasonality of precipitation or well-drained, sandy soils limit the occurrence of more mesic species (Nowacki and Abrams, 2008). Likewise, pine-oak forests are maintained in areas where fire is prevalent either naturally or as a management strategy (Parshall et al., 2003; Neill et al., 2007; Scheller et al., 2008) with pines being more successful under shorter fire return intervals and oaks being successful if fire is excluded from the system (Scheller et al., 2008). Even though oaks and pines inhabit many of the same ecosystems, they differ in terms of leaf type and hydraulic systems with oaks having larger leaves that are deciduous in many species and relying on large-diameter vessels with high hydraulic conductivity for water transport. Pines, on the other hand, have small, evergreen needle-like leaves and rely on smaller diameter tracheids for water transport. These large differences in leaf habit and water transport capacity of co-occurring oaks and pines could suggest that they have different strategies for obtaining and using nutrients and water, particularly in ecosystems with sandy soils that exhibit low water- and nutrient-holding capacity in the upper soil layers.

The sandy soils of the New Jersey Pinelands on the Atlantic Coastal Plain provide the ideal opportunity to compare strategies of water and nutrient use between oaks and pines in a resource-limited ecosystem that may result from their differences in leaf morphology, leaf habit and water transport properties. In particular, using both water and nutrients efficiently should be important for oaks and pines in ecosystems where resources are limiting. The edaphic features of this ecosystem also provide the opportunity to study tradeoffs between using water or using nutrients efficiently both within each genus and between genera. Water-use efficiency (WUE) and photosynthetic nitrogen-use efficiency (PNUE) describe the amount of water transpired or N content per unit leaf area, respectively, for a given rate of photosynthetic assimilation. Several studies have found an inverse relationship between PNUE and WUE (Field et al., 1983) across species within a common ecosystem (DeLucia and Schlesinger, 1991), across tree size (Nabeshima and Hiura, 2004), or along a latitudinal gradient (Sheng et al., 2011). Indeed, we would expect trees to experience a trade-off between water- and nitrogen-use efficiency in ecosystems with sandy soils because a reliable source of water will be found in the deeper soil layers while much of the available nitrogen will be located near the surface in the decomposing litter layer. This spatial segregation of resources could mean that optimizing uptake of one resource occurs at the expense of the other. Likewise, stomatal conductance, which affects the rate of carbon dioxide influx into leaves and evaporation of water vapor out of leaves, provides a link between WUE and PNUE. Individuals with lower stomatal conductance may have higher WUE, but the lower concentration of carbon dioxide within leaves may decrease PNUE. Alternatively, decreased nitrogen content in leaves may lead to higher PNUE while making stomatal conductance less efficient, thereby decreasing WUE. Therefore, both WUE and PNUE are important for trees to achieve optimal productivity in resource-limited environments with each determined by both resource availability and competitive acquisition rates of each tree type.

The anatomical and physiological differences between oaks and pines could suggest that these tree types differ in resource acquisition strategies for water and nutrients. In terms of hydraulic architecture, McCulloh et al. (2010) found that, among various tree types, ring-porous species like oaks had the highest leaf-specific hydraulic conductivity, the fewest vessels per unit sapwood area and the greatest taper in their vascular network (which aids in offsetting effects of increasing path length as trees grow taller) while conifers, like pines, had the lowest leaf-specific conductivity, the highest proportion of conduits per unit sapwood area and the lowest taper in the vascular network, making water transport more size-dependent. However, Poyatos et al. (2008) found that *Pinus sylvestris* (L.) had higher leaf specific hydraulic conductance than co-occurring *Quercus pubescens* (Willd.) growing in a Mediterranean climate. In terms of stomatal regulation of water transport, in a global synthesis study, Choat et al. (2012) found that conifers had the largest “safety margin” between minimum *in situ* water potential and the water potential at which 50% of hydraulic conductivity is lost to embolism. On the other hand, several studies have found that oaks and other ring-porous species tend to be more anisohydric, exhibiting increasingly more negative leaf water potentials with increasing evaporative demand and drought stress (Cavender-Bares and Bazzaz, 2000; Zweifel et al., 2007; Poyatos et al., 2008; Klein et al., 2013; Meinzer et al., 2013). However, in a synthesis study, Martínez-Vilalta et al. (2014) sought to use the empirical relationship between predawn and midday leaf water potentials to directly compare isohydric/anisohydric behavior across species and ecosystem types and found that oaks and pines had similar behavior [Supplementary Material; Martínez-Vilalta et al. (2014)] with both genera considered “partial isohydric.” In terms of nutrient acquisition, oaks tend to have higher foliar N concentrations than pines within the same ecosystem (Bockheim and Leide, 1991; Kolb and Stone, 2000; García-Barrios and González-Espinosa, 2004). Also, based on tradeoffs between leaf lifespan and leaf mass per unit area (LMA) vs. productivity and nutrient content, evergreen pines should have higher LMA, lower productivity and lower nutrient content than shorter-lived deciduous oak leaves (Wright et al., 2004). Therefore, although differences in anatomy and leaf habit are evident between pines and oaks, results from previous studies are mixed with regard to how these differences will affect physiological functioning of each genus in a resource-limited environment.

This study sought to compare resource use and efficiency as well as stomatal responses to environmental drivers of the dominant oak and pine species in the New Jersey Pinelands National Reserve located within the Atlantic Coastal Plain. The study area consists of sandy soils that tend to be excessively well-drained having low water holding capacity and nutrient retention. Due to these conditions, both WUE and PNUE should be important in order for species to successfully compete and our objective was to compare resource acquisition and resource use efficiency between oaks and pines to determine the trade-offs in resource-use strategies both within each genus and when comparing across genera. Likewise, because stomatal conductance is one of the controlling factors in both WUE and PNUE, our second objective was to determine the relationships between canopy stomatal conductance and its environmental controls including vapor pressure deficit (VPD), soil moisture and photosynthetic photon flux density (PPFD) in oaks and pines. Specifically, we hypothesize that (1) pines, with their evergreen leaf habit and water transport system that is reliant on small tracheids, will exhibit higher PNUE and WUE compared to oaks and (2) oaks will exhibit greater stomatal conductances than pines and will be less responsive to environmental conditions in accordance with an anisohydric stomatal habit. A more complete understanding of photosynthetic capacity and stomatal dynamics in both oaks and pines will help to elucidate how oak-dominated, pine-dominated, and mixed forests function given environmental constraints, and will allow for more accurate modeling of forest carbon sequestration processes and water use given future climate change as well as changes in disturbance regimes.

## Materials and methods

### Site descriptions

**Figure 1:**
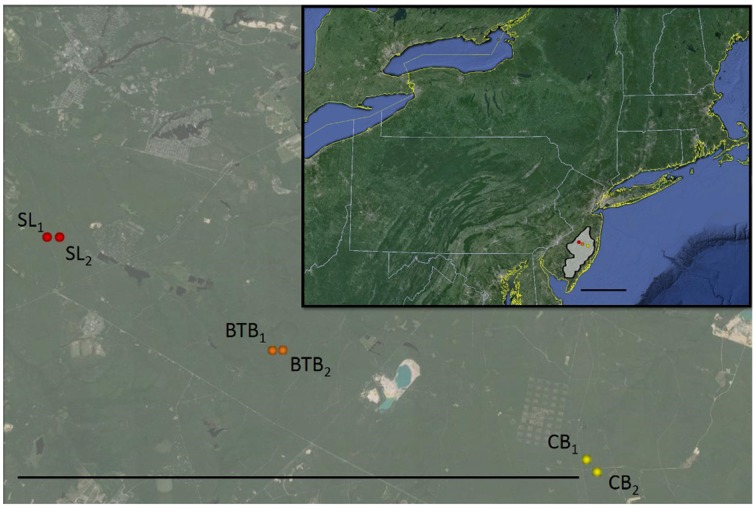
**Location of the New Jersey Pinelands National Reserve (in inset, shaded in gray; scale bar = 100 km) and locations of Silas Little (SL; in red), Brendan T Byrne (BTB; in orange), and Cedar Bridge (CB; in yellow) sites**. Scale bar on magnified map = 20 km. Map data: Image NOAA, Image Landsat, Data SIO, NOAA, U.S. Navy, NGA, GEBCO, © 2015 Google.

Sites were located within the 470,000 ha New Jersey Pinelands National Reserve (Figure 1) which includes upland forests composed of 46% oak-dominated forests, 31% oak-pine mixed forests, and 23% pine-dominated forests (Lathrop and Kaplan, 2004). Soils in the region are sandy and well-drained with limited nutrient-holding capacity and the site receives about 1100 mm of yearly precipitation. The site is cool temperate with average summer temperatures of approximately 22.7°C and average winter temperatures of approximately 1.3°C [www.ameriflux.ornl.gov; Clark et al. (2012)]. Due to the dry soil and litter layer conditions, wildfires tend to be prevalent (Little, 1979) and as a result, prescribed burning is routinely performed in late winter/early spring to reduce the risk of wildfires. Therefore, at each site, trees in both recently burned and unburned stands were pooled in order to capture the variability across the landscape due to the prescribed fire management. For more information on specific physiological differences resulting from prescribed fire, see Renninger et al. (2013). The summer growing season tends to be warm and humid with all months receiving moderate rainfall (June averages about 90 ± 6 mm of rainfall, July about 116 ± 7 mm and August about 130 ± 9 mm; www.ncdc.noaa.gov).

#### SL site

Measurements were made within the United States Department of Agriculture Forest Service Silas Little Experimental Forest (Rutgers Pinelands Research Station; 39° 51′ 57″ N, 74° 35′ 46″ W; referred to below as the “SL” site) in two stands (Figure 1), one of which was burned in a prescribed fire in March, 2012. This site is an oak-dominated stand with the dominant tree species consisting of mainly chestnut oak (*Quercus prinus* L.) and black oak (*Quercus velutina* Lam.) with scattered scarlet oak (*Quercus coccinea* Muenchh.), white oak (*Quercus alba* L.), post oak (*Quercus stellata* Wangenh.), pitch pine (*Pinus rigida* Mill.), and shortleaf pine (*Pinus echinata* Mill.). Maximum canopy height is approximately 19.5 m, stand age is 99 years and stand basal area is 17.6 m^2^ ha^−1^ (Renninger et al., 2014; Skowronski et al., 2014). This study focused on the predominant oak species in this forest (*Q. prinus*, *Q. velutina* and *Q. alba*). All *Quercus* spp. study individuals were measured at this site as well as overstory *Pinus* spp. individuals. The understory is composed of mainly huckleberry and blueberry shrubs (*Vaccinium* and *Gaylussacia* sp.), bracken fern (*Pteridium aquilinum* L. Kuhn), sedges, mosses, and lichens. This site was defoliated by gypsy moths (*Lymantria dispar* L.) in the summers of 2007 and 2008 and information about its effects can be found in Clark et al. (2012) and Schäfer et al. (2010). Depth to groundwater is approximately 7 m at this site (Schäfer et al., 2014; United States Geological Survey http://waterdata.usgs.gov/nwis/).

#### BTB site

*Pinus rigida* individuals were measured at two pine-dominated stands within the Brendan T. Byrne State Forest (Figure 1; 39° 53′ N, 74° 30′ W; subsequently referred to as the “BTB” site). One stand was burned in a prescribed fire in March, 2011. Both stands are composed primarily of pitch pine with scattered overstory oak species including white oak, chestnut oak and black oak. Maximum canopy height is approximately 12.5 m and stand basal area is about 26.9 m^2^ ha^−1^ (Renninger et al., 2013). The understory consists of scrub oaks (*Q. marilandica* Muenchh., *Q. ilicifolia* Wangenh.) blueberry and huckleberry shrubs, bracken fern, sedges, mosses, and lichens. Depth to groundwater is approximately 7.3 m at this site (United States Geological Survey site: 395234074302501 051587–Mb Up-2; http://waterdata.usgs.gov/nwis/).

#### CB site

*Pinus rigida* individuals were also measured at two stands located in the Greenwood Wildlife Management Area near the Cedar Bridge Fire Tower (Figure 1; 39° 49′ 4.19” N, 74° 22′ 32.28” W; subsequently referred to as the “CB” site). One stand was burned in a prescribed fire in March, 2013 (Clark et al., 2014). The stands are pine-dominated, with pitch pine in the overstory and white oak and scrub oaks in the understory. Maximum canopy height is approximately 12 m, stand age is 90 years, and basal area is 14.3 m^2^ ha^−1^ (Clark et al., 2012). The understory is also composed of blueberry and huckleberry shrubs, bracken fern, sedges, mosses and lichens. Depth to groundwater is approximately 21 m at this site (United States Geological Survey site: 394949074202901 290789–Cedar Brg Twr1; http://waterdata.usgs.gov/nwis/).

### Environmental data

**Figure 2:**
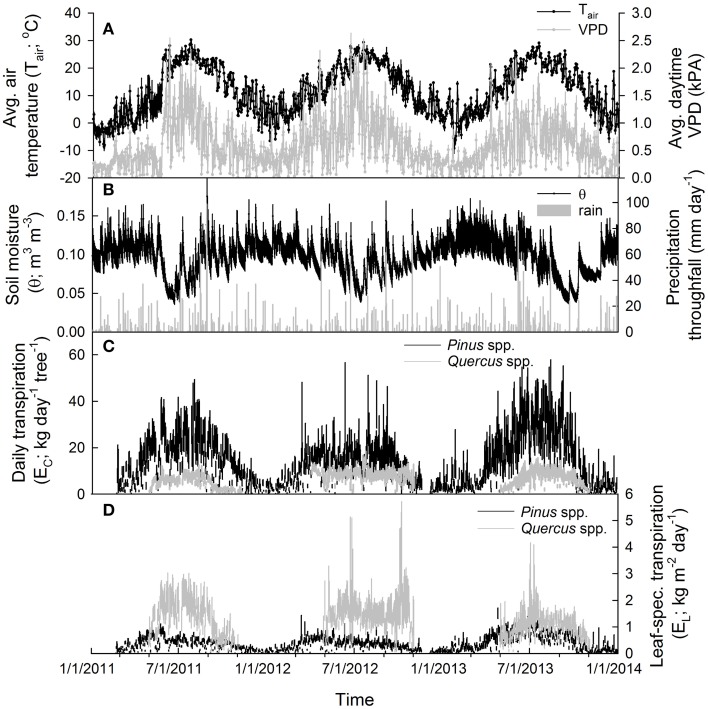
**Environmental and transpiration data (averaged across all sites) during the 2011–2013 study period, including (A) average daily air temperature (mean ± SE; °C) and average daytime vapor pressure deficit (VPD; mean ± SE; kPa) (B) average daily soil moisture (mean ± SE; m^3^ m^−3^) in the top 30 cm and total daily throughfall (mm day^−1^). (C)** Total daily tree-level transpiration (E_C_; kg day^−1^; mean ± SE of all individuals in each site/species category) for oaks and pines and **(D)** total daily leaf-specific transpiration (E_L_; kg m^−2^_leaf area_ day^−1^; mean ± SE of all individuals in each site/species category) for oaks and pines.

At each site, meteorological and edaphic parameters were measured throughout the study period (Figures 2A,B). Temperature and relative humidity were measured using Vaisala HMP45C sensors (Campbell Scientific Inc., Logan UT, USA) located in the mid-canopy of each forest and used to calculate VPD following Goff and Gratch (1946). Precipitation and throughfall were measured with Texas Electronics TE525M tipping buckets (Campbell Scientific Inc.) and soil moisture content in the upper 0.3 m of the soil was measured in four locations at each site using CS616 sensors (Campbell Scientific Inc.). All sensors were attached to dataloggers (Campbell Scientific Inc.) located in each site that collected data every 30 s and averaged data every 30 min. Photosynthetic photon flux density (PPFD) was measured at the SL and CB sites with LI-190SB quantum sensors (LI-COR Biosciences Inc., Lincoln NE, USA) located above each canopy on a tower (Clark et al., 2012).

### Leaf gas exchange

Leaf gas exchange was measured at the BTB site in 2011, 2012, and 2013 and at the SL and CB sites in 2012 and 2013 using a LI-COR 6400 XT photosynthesis system (LI-COR Biosciences Inc.) with a red/blue light source attached. For each individual during each measurement period, both light response and net assimilation to internal CO_2_ concentration (*A/C_i_*) curves were performed. Measurements for each year were made in July after the youngest cohort of needles on the *Pinus* spp. was fully expanded. Six *Pinus* spp. individuals were measured in each site during each yearly measurement cycle. For oaks in summer 2012, eight *Q. alba*, eight *Q. prinus*, and nine *Q. velutina* individuals were measured, and in 2013, six *Q. alba*, six *Q. prinus*, and six *Q. velutina* individuals were measured. Measurements were performed in mid-morning (after any dew had evaporated from leaves) to early afternoon (before significant moisture stress) on sunny days. The average temperature across measurement days was 26.8 ± 0.7°C. Humidity levels in the chamber were not controlled unless they became excessive and generally did not differ by more than 10% from ambient relative humidity measured at each site. Averaged across all curves measured, chamber vapor pressures deficits were about 2.6 ± 0.09 kPa, temperatures were about 28.1 ± 0.5°C and relative humidities were about 42.5 ± 1.5%. Because crowns could not be accessed directly, stems were cut with a pole pruner, recut under water to remove embolisms, and kept in a container of water located in a sunny understory gap while measurements were being made. Curves were measured within 5 min of cutting branches from trees in order to minimize the effects of measuring gas exchange on cut branches. Likewise, stomatal conductances in this study were higher than values reported in an earlier study on these oak species when leaves were accessed directly via a canopy lift (Schäfer, 2011). For oaks, the entire leaf chamber was filled with leaf tissue, while for pines, one or two fascicles (3–6 needles) were placed in the leaf chamber for gas exchange measurements. Chamber leaf areas for pines were then adjusted by collecting all leaves inside the chamber, scanning them using a flatbed scanner (Epson V30, Long Beach, CA) with a scaling factor and determining their single-sided area using Image J software (Scion Image, Frederick, MD, USA).

Light response curves were produced by holding CO_2_ concentrations in the leaf chamber constant at 400 ppm and varying light levels from 1500 to 0 μmol photons m^−2^ s^−1^. Light response curves took, on average, 11.6 ± 1.0 min to complete with about 86 ± 6 s for photosynthetic rates to equilibrate after each light level change. Data were then fitted with non-rectangular hyperbola equations developed by Prioul and Chartier (1977) and curve-fitting routines developed by Lobo et al. (2013). When non-rectangular hyperbola equations did not provide a satisfactory fit, exponential equations were used instead (Lobo et al., 2013). From the fitted equations, parameters describing the light response curves were derived including A_max_ (maximum photosynthetic assimilation rate), light compensation point (the light required for photosynthetic assimilation to balance respiration), quantum yield (the initial linear increase in photosynthetic assimilation with increasing light) and dark respiration rate (rate of CO_2_ production in darkness).

To produce *A*/*C*_i_ curves, light levels were held constant at 1500 μmol m^−2^ s^−1^ and CO_2_ concentrations in the leaf chamber began at 400 ppm, were progressively dropped to 50 ppm, returned to 400 ppm and raised by 200 ppm increments until photosynthetic rates saturated. *A*/*C*_i_ curves took, on average, 13.4 ± 2.1 min to complete with about 102 ± 3 s for photosynthetic rates to equilibrate after each change in CO_2_ concentration. Leaf-level stomatal conductance (g_s_; mol H_2_O m^−2^ s^−1^) and instantaneous ratios of internal CO_2_ concentration to ambient CO_2_ concentration (c_i_/c_a inst._) were estimated from data used to create the light response and *A*/*C*_i_ curves during saturating light levels (1500 μmol m^−2^ s^−1^) and ambient CO_2_ levels (400 ppm) in the leaf chamber. Also, instantaneous water use efficiency (WUE_inst._; μ mol CO_2_ mmol^−1^ H_2_O) was calculated by dividing corresponding net photosynthetic assimilation (*A*_net_; μ mol CO_2_ m^−2^ s^−1^) by transpiration (*E*; mmol H_2_0 m^−2^ s^−1^) and instantaneous intrinsic water use efficiency (iWUE_inst.;_ μ mol CO_2_ mol^−1^ H_2_O) was calculated by dividing net assimilation by stomatal conductance at saturating light and ambient CO_2_.

Data from *A*/*C*_i_ curves were used to calculate maximum Rubisco-limited carboxylation rates (*V*_Cmax_), maximum electron transport-limited carboxylation rates (*J*_max_), carboxylation rates limited by triose phosphate utilization (TPU), and daytime respiration rates (*R*_day_). Non-linear curves based on theory and equations from Farquhar et al. (1980) were fitted to the data after defining the Rubisco-limited, electron transport-limited and triose phosphate utilization-limited portions of the *A*/*C*_i_ curves (Sharkey et al., 2007). Curve-fitting routines developed by Sharkey et al. (2007) were used to calculate temperature-adjusted (to 25°C) parameter estimates that minimized the sums of squares error between measured and modeled data, thereby providing estimates of *V_C_*_max25_, *J*_max25_, TPU_25_, and *R*_day25_. Data from *A*/*C*_i_ curves were also used to estimate the Ball-Berry parameter (Leuning, 1990; Collatz et al., 1991) which is the slope (m) of the following equation:

(1) $$g_{s}=m\;\times \frac{A\times rh_{s}}{C_{\text{S}}-\Gamma }+g_{o}$$

where *rh_s_* is relative humidity at the leaf surface, *C*_s_ is CO_2_ concentration at the leaf surface (μ mol mol^−1^), Γ is the CO_2_ compensation point (μ mol mol^−1^) of the corresponding *A/C*_i_ curve and *g*_o_ is minimum conductance (mol H_2_O m^−2^ s^−1^). Γ was calculated as the x-intercept of a linear regression fitted to the initial points on each *A/C*_i_ curve.

### Leaf nutrient and isotopic composition

After photosynthetic measurements were made, leaves were returned to the lab and their areas estimated by scanning them and using image analysis software (see above). Leaves were then dried at 70°C for at least 3 days and weighed in order to calculate leaf dry mass per unit area (LMA; g m^−2^). After weighing, leaves were ground finely using a ball mill, sealed in aluminum capsules and their carbon isotopic ratios (δ^13^C), carbon and nitrogen concentrations determined (Duke University Stable Isotope facility DEVIL, Durham, NC; UC Davis Stable Isotope facility, Davis, CA). Carbon isotope discrimination (Δ; ‰) was calculated from the carbon stable isotope data using the following equation (Farquhar et al., 1989):

(2) $$\Delta =\left(\frac{\delta^{13}a-\delta^{13}C}{1000+\delta^{13}C}\right)*1000$$

where δ^13^a is the isotopic concentration of the source air measured at the SL site by a Picarro G1101-I Isotopic CO_2_ Analyzer (Picarro Inc. Santa Clara, CA, USA) sampling air at the top of the 19 m tall canopy tower, 10 m above ground (at the point of maximum canopy leaf area) and 0.5 m above ground (data obtained from http://www.nrs.fs.fed.us/data/climate-tower/). Carbon isotopic ratios of source air averaged −9.4‰ during the first part of the 2011 growing season (May-July), −11.4‰ during the first part of the 2012 growing season and −10.4‰ during the first part of the 2013 growing season. Intrinsic water use efficiency (iWUE_iso._) and integrated c_i_/c_a_ ratios (c_i_/c_a iso._) were also estimated from the isotope data as follows (Farquhar et al., 1989):

(3) $$iWUE_{iso.}=\;\frac{c_{a}}{1.6}\times \left(\frac{27-\Delta }{27-4.4}\right)$$

(4) $$\frac{c_{i}}{c_{a}}iso.=\;\frac{\Delta -4.4}{27-4.4}$$

where c_a_ is the ambient CO_2_ concentration (400 ppm), 1.6 is the ratio of water vapor to carbon dioxide diffusivity, 27‰ is the discrimination of Rubisco to ^13^C and 4.4‰ is the diffusive discrimination of ^13^C in air through the stomata.

Leaf N concentrations were multiplied by LMA to scale N per unit leaf area (N_area_). Photosynthetic nitrogen use efficiency (PNUE; μ mol CO_2_ g^−1^ N s^−1^) was calculated by dividing net photosynthetic assimilation rates at ambient CO_2_ and saturating light conditions by the N_area_ (Field et al., 1983).

### Sap flow, canopy transpiration and canopy stomatal conductance

In 2011, sap flow rates were measured in seven *Q. prinus*, five *Q. velutina*, and one *P. echinata* at the SL site and 19 *P. rigida* at the BTB site. In 2012, sap flow rates were measured in four *Q. alba*, 11 *Q. prinus*, 13 *Q. velutina*, one *P. echinata*, and five *P. rigida* at the SL site, 19 *P. rigida* individuals at the BTB site and eight *P. rigida* at the CB site. In 2013, sap flow rates were measured in four *Q. alba*, 11 *Q. prinus*, 13 *Q. velutina*, one *P. echinata*, and five *P. rigida* at the SL site and 18 *P. rigida* individuals at the CB site. *P. rigida* and *P. echinata* were combined in the analyses as they have similar properties and occasionally, naturally hybridize with one another (Ledig and Little, 1979).

Sap flow rates were measured using Granier-style heat dissipation sensors (Granier, 1987). Sensors consisted of a pair of hypodermic needles with a thermocouple inside each that were radially inserted into the sapwood. One of the sensors was constantly heated and located 0.1 m above the unheated reference sensor. *Quercus* spp. received 1 cm long sensors as they have shallow sapwood and *Pinus* spp. received 2 cm long sensors. *Pinus* spp. individuals larger than 30 cm diameter breast height (DBH) had an outer (0–2 cm depth) sapwood sensor and an inner sapwood sensor (2–4 cm depth) to capture radial patterns in sap flow. Aluminum shields were placed over the sensor pairs to protect against sunflecks and sensors were connected to dataloggers and AM16/32 multiplexers located at each site (Campbell Scientific Inc.) that measured the mV output of the sensor pairs (proportional to the temperature difference between the heated and reference sensor) every 30 s and averaged data every 30 min. An empirical equation was used to convert mV values to sap flow rates (*J*_S_; kg m^−2^ s^−1^) as follows (Granier, 1987):

(5) $$J_{s}=0.119\times {\left[\frac{\Delta T_{max}}{\Delta T}-1\right]}^{1.23}$$

where Δ*T*_max_ is the temperature difference between sensors when no water is flowing and Δ*T* is the temperature difference when sap flow is occurring. To determine zero flow conditions for ΔT_max_, data were chosen when VPD <0.05 kPa over a 2 h period and ΔT_max_ values were stable over a 2 h period (Oishi et al., 2008). Additionally, because *Quercus* spp. are ring-porous and sap flow measured with heat dissipation sensors tends to be underestimated (Taneda and Sperry, 2008; Bush et al., 2010; Hultine et al., 2010), an additional correction was applied to the sap flow data as described in Renninger and Schäfer (2012).

Individual whole-canopy transpiration rates (E_C_; kg day^−1^) were calculated by multiplying half-hourly sap flow rates by the sapwood area (*A*_S_, m^2^) of the individual (Figure 2C). For oaks, relationships between DBH and A_S_ were made by coring selected individuals and determining sapwood depth via a color change between sapwood and heartwood (Phillips et al., 2003). For all measured *Quercus* spp., the following sigmoidal equation (*r*^2^ = 0.88) was used to calculate A_S_:

(6) $$A_{S}\left(m^{2}\right)=\frac{0.0188}{1-e^{-\left(\frac{DBH(cm)\;-\;20.97}{4.791}\right)}}$$

An equation for calculating A_S_ from DBH for *Pinus* spp. has been previously reported in Renninger et al. (2013) and was used in this study as well. Leaf-specific transpiration rates (*E*_L_; kg m^−2^_leaf area_ day^−1^) were calculated by dividing sap flow per unit A_S_ by the leaf area of each individual (Figure 2D). For oaks, leaf areas were estimated in 2012 using an LAI2000 leaf area meter (LI-COR Biosciences Inc.). Specific methodologies are described in Renninger et al. (2014). Briefly, light transmittance was measured at dawn at four locations under each tree and compared with a sensor measuring clear sky conditions to estimate leaf density (leaf area/canopy volume). Canopy profiles were measured on two planes for each tree in order to calculate canopy volume. Trees were sufficiently spaced so that canopies did not overlap with one another. Leaf areas in 2011 and 2013 were calculated from corresponding A_S_ assuming that leaf area to sapwood area ratios remained constant throughout the study period (Rogers and Hinckley, 1979). For pines, leaf areas were calculated using an allometric equation reported in Whittaker and Woodwell (1968) that was developed for *Pinus rigida* growing on sandy soils in Long Island, NY, USA. Because this equation calculates all-sided leaf areas for *Pinus* spp., a single-sided leaf area was calculated by dividing all-sided leaf areas by π (Vose et al., 1994).

Mean canopy stomatal conductance to water vapor (*G*_S_; m s^−1^) was also estimated from the half-hourly *E*_L_data as follows (Köstner et al., 1992; Ewers et al., 2001):

(7) $$G_{s}=\frac{K_{G}*E_{L}}{VPD}$$

where *K*_G_ is a conductance coefficient [115.8 + 0.4236*air temperature in °C; Phillips and Oren (1998)]. In order to convert mean canopy stomatal conductance in m s^−1^ to mol H_2_O m^−2^ s^−1^, *G*_S_ was divided by the density of water vapor $\left({\text{m}}^{3}/\text{mol},0.0224*\frac{air\;temp.(K)}{273}\right)$. These equations require that boundary layer conductance is high, leaf temperature and air temperature are similar, VPD is constant throughout the canopy and there is negligible use of stored bole water. Forests in the New Jersey Pinelands are relatively open and individual tree crowns have low leaf area densities, therefore individual leaves are assumed to be coupled with the surrounding atmosphere and air within the canopy is assumed to be well-mixed. For oaks, sapwood volumes are relatively small, and therefore, stored bole water use is negligible (Renninger et al., 2014). For pines, lag analysis between bole sap flow and VPD showed that stored bole water did not contribute significantly to transpiration (Phillips et al., 1997). Once half-hourly *G*_S_ was calculated, average daytime values were obtained by averaging all half-hourly *G*_S_ corresponding to half-hourly VPD above 0.5 kPa (Ewers et al., 2001). For each individual, average daytime G_S_ values were plotted vs. the natural log of VPD and linear regressions fitted to these data. The negative slope of this relationship (representing stomatal sensitivity to VPD; δG_S_/δ lnVPD) and the y intercept (representing reference G_S_ at VPD = 1 kPa; G_Sref;_ Oren et al., 1999) were recorded. The ratio of stomatal sensitivity to VPD and G_Sref_ was then calculated which represents a stomatal sensitivity normalized by reference stomatal conductance (“stomatal sensitivity ratio”).

### Data analysis and statistics

Leaf-level gas exchange and composition parameters were compared between oaks and pines using linear mixed effects models in R version 3.1.2 [lme function; Pinheiro et al. (2014)] with “tree within site” included as a random effect in each model. *Post-hoc* tests were also performed to compare parameters across oak species and pines across the three sites using the general linear hypotheses test [glht function in the multcomp package; Hothorn et al. (2008)] with Tukey contrasts in R (Tables S1, S2). Regressions were plotted and estimates of r^2^ and slope *p*-values were performed in Sigmaplot (SPSS Inc., Chicago, IL, USA). Analysis of covariance (ANCOVA) was performed in R using the aov function to compare slopes of linear regression relationships between oaks and pines. In order to determine if stomata responded differently to changes in VPD across a soil moisture or energy availability (represented by PPFD) gradient, mean daytime G_S_ data were sorted by either relative soil moisture content or daily integrated PPFD (mol m^−2^ d^−1^) and grouped under similar daily soil moisture or light conditions. Relative soil moisture content was calculated as soil moisture content divided by the highest soil moisture content measured in each study site (assumed to be field capacity). In each soil moisture or daytime PPFD category, mean daytime G_S_ was plotted vs. natural log-transformed mean daytime VPD and linear regressions fitted to the data. The slope of these regression equations (δG_S_/δ lnVPD) represents canopy stomatal sensitivity to VPD (Oren et al., 1999) and these slope parameters were plotted across a soil moisture or daytime PPFD gradient to determine how stomatal sensitivity to VPD was affected by soil moisture deficits and energy availability.

## Results

### Between year and between site environmental variation

Daytime temperatures were, on average, 25.0 ± 0.3°C in 2011, 24.9 ± 0.4°C in 2012 and 24.4 ± 0.3°C in 2013 during each growing season (June–Aug.) and average daytime VPD was 1.27 ± 0.05 kPa in 2011, 1.28 ±0.06 kPa in 2012 and 0.98 ± 0.04 kPa in 2013 during each summer growing season (Figure 2). In 2011, the sites received about 1290 mm of throughfall and soil moisture in the upper 30 cm during the summer averaged about 0.06 ± 0.002 m^3^ m^−3^ at the SL site. In 2012, the sites received about 1050 mm of throughfall and soil moisture averaged about 0.06 ± 0.001 m^3^ m^−3^ at the SL site. In 2013, the sites received about 1210 mm of throughfall and soil moisture averaged about 0.07 ± 0.002 m^3^ m^−3^ at the SL site (Figure 2). For maximum daytime photosynthetic photon flux density (PPFD), 2011 and 2012 had similar averages over the summer growing season with 1680 ± 33 μmol m^−2^ s^−1^ and 1681 ± 36 μmol m^−2^ s^−1^ respectively while 2013 was slightly lower with 1671 ± 39 μmol m^−2^ s^−1^. Between sites, daily soil moisture in the upper 30 cm during the 2012 summer growing season averaged about 0.06 ± 0.002 m^3^ m^−3^ at the SL site, 0.08 ± 0.002 m^3^ m^−3^ at the BTB site and 0.10 ± 0.003 m^3^ m^−3^ at the CB site. Therefore, 2011 and 2012 were similar in terms of environmental conditions with 2013 exhibiting less atmospheric and soil moisture deficits compared with the prior years. Between sites, soil moisture in the upper layers was generally higher with increasing proximity to the ocean while depth to the groundwater also increased in the sites from west to east (Figure 1).

### Leaf-level gas exchange

**Figure 3:**
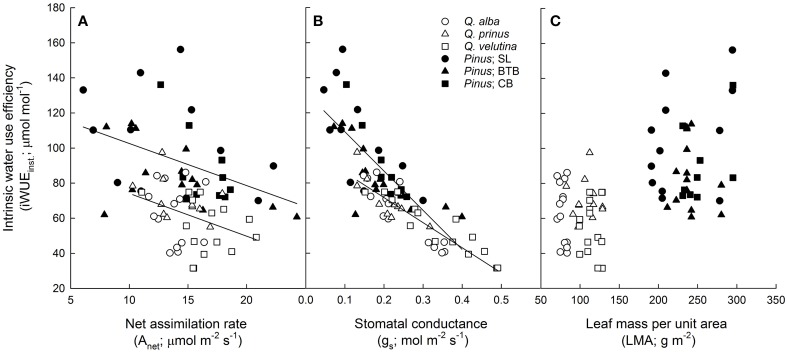
**Instantaneous intrinsic water use efficiency (iWUE_**inst**._; μ mol CO_2_ mol^−1^ H_2_O) vs. (A) net photosynthetic assimilation rate (A_**net**_; μ mol m^−2^ s^−1^) for oaks (*y* = −2.4x + 98.5; *r*^2^ = 0.13) and pines (*y* = −2.4x + 126.6; *r*^2^ = 0.19), (B) stomatal conductance (g_s_; mol H_2_O m^−2^ s^−1^) for oaks (*y* = −143x + 100.3; *r*^2^ = 0.79) and pines (*y* = −224x + 131.6; *r*^2^ = 0.54), and (C) leaf mass per unit area (LMA; g m^−2^) for oaks and pines**. Points represent average across *A*/*C*_i_ and light response curves for each individual during each measurement period. Solid lines represent significant relationships (slope *P* < 0.05).

All photosynthetic parameters describing the light response and *A*/*C*_i_ curves were statistically similar (across sites and species) between oak and pine foliage (Table 1). Across oak species, *Q. velutina* had statistically higher A_max_ rates than *Q. alba* and *Q. prinus* as well as higher V_Cmax_, J_max_, TPU and daytime respiration rates compared to the other oak species (Table S1). Across sites, parameters estimated from light response and *A*/*C*_i_ curves were similar for pines except daytime respiration rates which were significantly higher in the SL site than the other two sites (Table S2). Oak foliage had significantly higher transpiration rates (E), stomatal conductances (g_s_) and c_i_/c_a inst_ ratios based on gas exchange compared with pine foliage, and therefore had significantly lower water use efficiency (WUE_inst._) and intrinsic water use efficiency (iWUE_inst._; Table 1). Across oak species, *Q. velutina* tended to have the largest transpiration, stomatal conductance and c_i_/c_a inst_ ratios, but species exhibited similar WUE (although *Q. alba* had a significantly higher intrinsic iWUE_inst_; Table S1). Across sites, pines at the SL site had significantly lower transpiration rates and ci/c_a inst_ ratios compared to the other two sites, but had the highest WUE (although iWUE_inst_ was not significantly different across sites; Table S2). For the physiological components that comprise instantaneous intrinsic water use efficiency, both oaks and pines displayed similar negative slope terms (*P* = 0.25) in the relationship between net photosynthetic assimilation rates (A_net_) and iWUE_inst._ (Figure 3A; *r*^2^ = 0.13 and 0.19 for oaks and pines respectively) although pines displayed a wider range of A_net_ values. Likewise, both oaks and pines had a statistically similar (*P* = 0.46) negative relationship between stomatal conductance and iWUE_inst._ (Figure 3B; *r*^2^ = 0.79 and 0.54 for oaks and pines respectively) with pines having lower stomatal conductances but higher LMA than oaks (Figure 3C). The Ball-Berry parameter, which describes the rate of change in leaf-level stomatal conductance with assimilation rate, leaf surface CO_2_ concentration and leaf surface humidity was also statistically similar between oak and pine foliage (Table 1).

**Table 1:** **Means and standard error (in parentheses) of leaf-level gas exchange, isotope and nutrient parameters across all measured oak and pine individuals**.

	***Quercus* spp**.	***Pinus* spp**.	***P*–value**
Maximum assimilation rate (A_max_; μ mol m^−2^ s^−1^)	16.2 (0.5)	16.4 (0.8)	0.99
Quantum yield (μ mol μ mol^−1^)	0.049 (0.002)	0.047 (0.003)	0.92
Light compensation pt. (μ mol m^−2^ s^−1^)	21.7 (2.3)	21.3 (2.8)	0.69
Dark respiration rate (μ mol m^−2^ s^−1^)	0.97 (0.10)	0.92 (0.13)	0.63
V_Cmax,25_ (μ mol m^−2^ s^−1^)^a^	85.4 (5.8)	83.1 (4.9)	0.82
J_max,25_ (μ mol m^−2^ s^−1^)^b^	100.5 (9.4)	95.1 (7.1)	0.78
TPU_,25_ (μ mol m^−2^ s^−1^)^c^	6.8 (0.6)	6.9 (0.5)	0.48
Daytime respiration rate (μ mol m^−2^ s^−1^)	3.8 (0.7)	4.4 (0.5)	0.09
Transpiration (E; mmol m^−2^ s^−1^)	5.16 (0.31)	4.70 (0.28)	**0.02**
Stomatal conductance (g_s_; mol m^−2^ s^−1^)	0.26 (0.02)	0.18 (0.01)	**0.001**
c_i_/c_a inst._^d^	0.70 (0.01)	0.62 (0.012)	**<0.001**
WUE_inst._ (A/E; μ mol mmol^−1^)^e^	3.49 (0.20)	3.59 (0.30)	**0.03**
iWUE_inst_. (A/g_s_; μ mol mol^−1^)^f^	63.2 (2.3)	92.4 (4.7)	**0.02**
Ball-Berry parameter (m)	7.8 (0.8)	5.4 (1.3)	0.08
δ ^13^C (‰)^g^	−29.7 (0.2)	−30.3 (0.2)	0.18
Δ(‰)^h^	19.1 (0.2)	20.4 (0.3)	0.17
c_i_/c_a iso._^i^	0.65 (0.008)	0.71 (0.01)	0.17
iWUE_iso._ (μ mol mol^−1^)^j^	87.8 (2.0)	73.5 (3.1)	0.17
Leaf mass per area (LMA; g m^−2^)	100.4 (3.7)	240.7 (7.3)	**<0.001**
Leaf nitrogen concentration (N; %)	2.16 (0.04)	1.03 (0.03)	**<0.001**
Leaf carbon concentration (C; %)	48.4 (0.5)	48.2 (0.4)	0.75
Leaf C/N ratio	22.6 (0.3)	47.6 (1.2)	**<0.001**
Nitrogen per unit leaf area (N_area_; g m^−2^)	2.17 (0.07)	2.47 (0.09)	**0.006**
PNUE (μ mol g^−1^ s^−1^)^k^	7.07 (0.19)	6.16 (0.40)	**0.01**

*Bold P-values denote significance at α < 0.05*.

a *Rubisco-limited carboxylation rate at 25°C*.

b *Electron transport-limited carboxylation rate at 25°C*.

c *Triose phosphate utilization-limited carboxylation rate at 25°C*.

d *Instantaneous ratio of [CO_2_]_inside leaf_ to [CO_2_]_ambient air_*.

e *Instantenous water-use efficiency*.

f *Instantenous intrinsic water–use efficiency*.

g *Leaf isotopic ratio*.

h *Leaf isotopic discrimination*.

i *Ratio of [CO_2_]_inside leaf_ to [CO_2_]_ambient air_ based on carbon isotope discrimination*.

j *Intrinsic water-use efficiency based on carbon isotope discrimination*.

k *Photosynthetic nitrogen-use efficiency*.

### Leaf isotope and nutrient content

**Figure 4:**
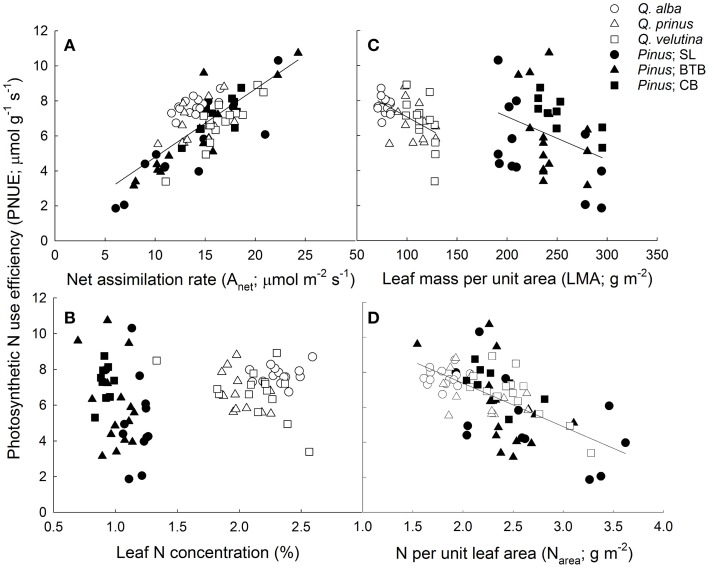
**Photosynthetic nitrogen use efficiency (PNUE; μ mol CO_2_ g^−1^ N s^−1^) vs. (A) net photosynthetic assimilation rate (A_net_; μ mol CO_2_ m^−2^ s^−1^; *y* = 0.39x + 0.89; *r*^2^ = 0.58) for oaks and pines, (B) leaf N concentration (%), (C) leaf mass per unit area (LMA; g m^−2^) for oaks (*y* = −0.032x + 10.2; *r*^2^ = 0.3) and pines (*y* = −0.027x + 12.0; *r*^2^ = 0.12), and (D) N per unit area (N_area_; g N m^−2^) for oaks and pines (*y* = −2.48x + 12.3; *r*^2^ = 0.38)**. Points represent average across *A*/*C*_i_ and light response curves for each individual during each measurement period. Solid lines represent significant relationships (slope *P* < 0.05).

**Figure 5:**
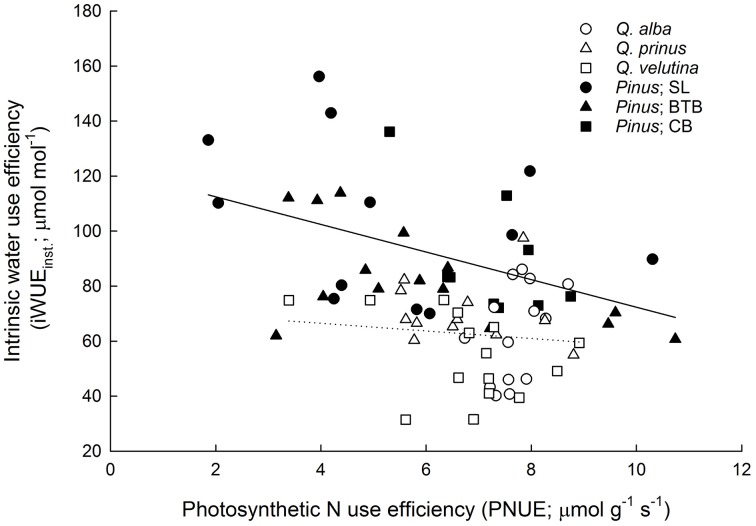
**Photosynthetic nitrogen use efficiency (PNUE; μ mol CO_2_ g^−1^ N s^−1^) vs. instantaneous intrinsic water use efficiency (iWUE_**inst**._; μ mol CO_2_ mol^−1^ H_2_O) for oaks and pines**. Pines exhibited a significant negative relationship (solid line; *y* = −5.02x + 122.5; *r*^2^ = 0.21) between PNUE and iWUE_inst._, while the slope of the relationship for oak was not statistically different from zero (dotted line; *P* = 0.54). Points represent average across *A*/*C*_i_ and light response curves for each individual during each measurement period. Solid lines represent significant relationships (slope *P* < 0.05); dotted lines represent non-significant relationships (slope *P* > 0.05).

Although pines had significantly greater water use efficiencies based on instantaneous leaf gas exchange, oaks, and pines were not significantly different in terms of intrinsic WUE based on carbon isotope data (iWUE_iso._; Table 1). Across species, oaks displayed similar iWUE_iso.,_whereas pines at the BTB site had significantly lower iWUE_iso._than pines at the SL or CB sites (Tables S1, S2). Pine needles exhibited over 2.5 times higher LMA compared with oak leaves, however oak leaves had about double the N concentrations and half the C/N ratios compared with pine needles (Table 1). Across oak species, *Q. alba* had significantly lower LMA and C/N ratios but higher leaf N concentration compared with *Q. prinus* and *Q. velutina* (Table S1). LMA did not differ between pines growing in different sites, but pines at the SL site had significantly higher leaf N concentrations and lower C/N ratios compared with pines growing at the BTB and CB sites (Table S2). When N concentrations were expressed per unit leaf area, pines had significantly higher N_area_ and significantly lower photosynthetic nitrogen use efficiencies (PNUE) compared with oaks (Table 1). Across oak species, *Q. alba* had significantly lower N_area_ but all species had similar PNUE (Table S1), while pines across sites had similar N_area_ and PNUE (Table S2). In terms of the physiological components that comprise PNUE, both oaks and pines displayed a statistically similar (*P* = 0.13) positive relationship between *A*_max_ and PNUE (Figure 4A; *r*^2^ = 0.58), no relationship between leaf N concentration and PNUE (Figure 4B; *P* = 0.20), statistically similar (*P* = 0.98) negative slope terms in the relationship between LMA and PNUE (Figure 4C; *r*^2^ = 0.30 and 0.12 for oaks and pines respectively), and a statistically similar (*P* = 0.31) negative relationship between N_area_ and PNUE (Figure 4D; *r*^2^ = 0.38). Pines exhibited a significant negative relationship between PNUE and iWUE_inst._ (*r*^2^ = 0.21, *P* = 0.006), while for oaks, the relationship was not significant (Figure 5).

### Transpiration and canopy stomatal conductance

Individual tree-level transpiration rates (E_C_) were, on average, about three times larger in pines compared to oaks during the growing season (Figure 2C; Table 2). Across sites, individual pine water use during the growing season was highest at the SL site with an average of 5200 kg tree^−1^ followed by pines at the CB site with 2700 kg tree^−1^ and pines at the BTB site with 2100 kg tree^−1^. Across oak species, *Q. prinus* had the highest individual tree water use with, on average, about 1300 kg tree^−1^, followed by *Q. velutina* with about 1100 kg tree^−1^ and *Q. alba* with about 880 kg tree^−1^. Therefore, at the pine-dominated stands (BTB and CB), individual tree water use in pines was about twice that of oaks but, at the oak-dominated (SL) site, more than four times greater in pines than oaks. On a yearly basis, pines transpired, on average, another 1200 kg tree^−1^ during the oak leaf-off period which is similar to the total yearly transpiration for oaks (Table 2). Because total growing season leaf areas for individuals are, on average, over five times higher for pines compared to oaks, average canopy leaf-specific transpiration rates (E_L_) were over 2.5 times greater for oaks compared to pines (Figure 2D; Table 2). Across sites, pines had similar E_L_ at the SL and CB sites averaging 0.58 ± 0.05 kg m^−2^ d^−1^ across years, but lower E_L_ at the BTB site (0.35 ± 0.03 kg m^−2^ d^−1^). For oaks at the SL site, *Q. velutina* had the highest E_L_ averaging 1.78 ± 0.3 kg m^−2^ d^−1^ across years, followed by *Q. prinus* (1.23 ± 0.1 kg m^−2^ d^−1^) and *Q. alba* (0.6 ± 0.06 kg m^−2^ d^−1^). Across sites for pines, E_L_ was highest in 2013 (0.6 ± 0.1 kg m^−2^ d^−1^) followed by 2011 (0.52 ± 0.07 kg m^−2^ d^−1^) and 2012 (0.4 ± 0.07 kg m^−2^ d^−1^; Figure 2). The opposite pattern was found for oaks across species with 2012 having the highest E_L_ (1.4 ± 0.5 kg m^−2^ d^−1^), followed by 2011 (1.2 ± 0.4 kg m^−2^ d^−1^) and 2013 (0.99 ± 0.2 kg m^−2^ d^−1^; Figure 2). Similar to E_L_, mean daytime canopy stomatal conductance (G_S_; mol m^−2^ s^−1^) measured when mean daytime VPD = 1 kPa was almost twice as high in oaks compared to pines (Table 2). Across sites, pines at the BTB site had the highest G_S_ at VPD = 1 kPa averaging 0.09 ± 0.003 mol m^−2^ s^−1^ across years, followed by pines at the SL site (0.08 ± 0.006 mol m^−2^ s^−1^) and pines at the CB site (0.06 ± 0.005 mol m^−2^ s^−1^). For oak species, *Q velutina* had the highest G_S_ at VPD = 1 kPa averaging 0.20 ± 0.009 mol m^−2^ s^−1^ across years, followed by *Q. prinus* (0.15 ± 0.007 mol m^−2^ s^−1^) and *Q. alba* (0.07 ± 0.003 mol m^−2^ s^−1^).

**Table 2:** **Means and standard error (in parentheses) of morphological and canopy-level water use properties for individual oaks and pines averaged across all years and study sites**.

	***Quercus* spp**.	***Pinus* spp**.	***P*–value**
Diameter at breast height (DBH; cm)	19.5 (0.9)	24.1 (1.3)	**0.006**
Crown area (m^2^)	7.9 (1.3)	10.7 (1.3)	0.15
Sapwood area (m^2^)	0.0086 (0.0008)	0.028 (0.003)	**<0.001**
Leaf area (m^2^)	8.3 (1.1)	45.9 (5.1)	**<0.001**
Growing season water use (E_C_; kg tree^−1^)	1200 (130)	3600 (660)	**<0.001**
Total yearly water use (E_C_; kg tree^−1^ year^−1^)	1200 (130)	4800 (900)	**<0.001**
Avg. daily leaf-specific transpiration (E_L_; kg m^−2^ day^−1^)	1.36 (0.14)	0.49 (0.03)	**<0.001**
Avg. daytime canopy stomatal conductance (G_S_; mol m^−2^ s^−1^)^*^	0.15 (0.006)	0.08 (0.002)	**<0.001**

*Bold P-values denote significance at α < 0.05*.

* *at average daytime vapor pressure deficit (VPD) = 1 kPa*.

**Figure 6:**
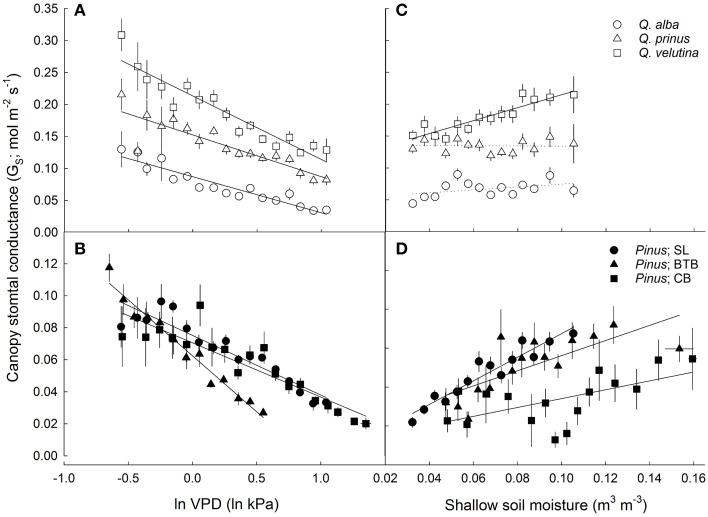
**Average daytime canopy stomatal conductance (G_S_; mol m^−2^ s^−1^; mean ± SE across all individuals in each site/species category) vs. natural log-transformed average daytime vapor pressure deficit (lnVPD; kPa) for (A) oaks including *Q. alba* (*y* = −0.056x + 0.087; *r*^2^ = 0.88), *Q. prinus* (*y* = −0.065x + 0.15; *r*^2^ = 0.78) and *Q. velutina* (*y* = −0.099x + 0.21; *r*^2^ = 0.89) and (B) pines at the SL site (*y* = −0.037x + 0.075; *r*^2^ = 0.88) the BTB site (*y* = −0.070x + 0.063; *r*^2^ = 0.97) and the CB site (*y* = −0.034x + 0.071; *r*^2^ = 0.82)**. G_S_ vs. soil moisture in the top 30 cm (m^3^ m^−3^) for **(C)** oaks including *Q. alba* (ns; *P* = 0.12), *Q. prinus* (ns; *P* = 0.54) and *Q. velutina* (*y* = 1.01x + 0.11; *r*^2^ = 0.84) and for **(D)** pines at the SL site (*y* = 0.61x + 0.027; *r*^2^ = 0.91), the BTB site (*y* = 0.39x + 0.037; *r*^2^ = 0.57) and the CB site (*y* = 0.23x + 0.031; *r*^2^ = 0.36). Solid lines represent significant relationships (slope *P* < 0.05); dotted lines represent non-significant relationships (slope *P* > 0.05).

**Figure 7:**
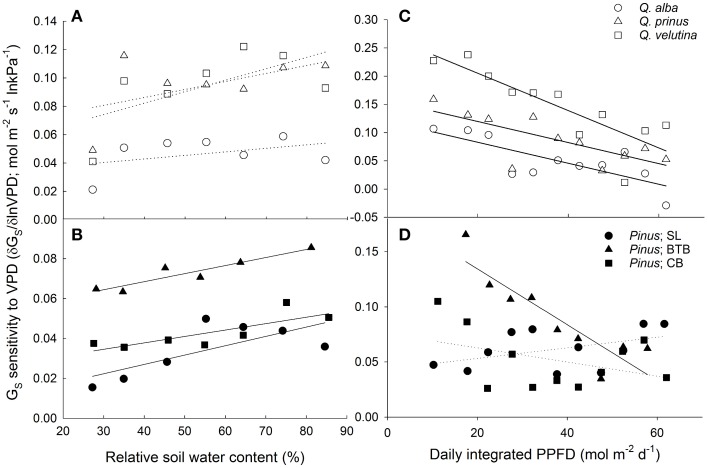
**Canopy stomatal conductance (G_S_) sensitivity to VPD (mol m^−2^ s^−1^ ln kPa^−1^) defined as the negative slope of the relationship between G_S_ and lnVPD vs. relative soil moisture content (%) for (A) oaks including *Q. alba* (ns; *P* = 0.36), *Q. prinus* (ns; *P* = 0.22) and *Q. velutina* (ns; *P* = 0.13) and for (B) pines at the SL site (*y* = 4.7× 10^−4^x + 0.0083; *r*^2^ = 0.53) the BTB site (*y* = 4.03× 10^−4^x + 0.0052; *r*^2^ = 0.87), and the CB site (*y* = 3.2× 10^−4^x + 0.025; *r*^2^ = 0.65)**. G_S_ sensitivity to VPD vs. daily integrated photosynthetic photon flux density (PPFD; mol _photons_ m^−2^ d^−1^) for **(C)** oaks including *Q. alba* (*y* = −0.0019x + 0.12; *r*^2^ = 0.59), *Q. prinus* (*y* = −0.0019x + 0.16; *r*^2^ = 0.54) and *Q. velutina* (*y* = −0.0033x + 0.27; *r*^2^ = 0.71) and for **(D)** pines at the SL site (ns; *P* = 0.17), the BTB site (*y* = −0.0025x + 0.19; *r*^2^ = 0.8) and the CB site (ns; *P* = 0.22). Solid lines represent significant relationships (slope *P* < 0.05); dotted lines represent non-significant relationships (slope *P* > 0.05).

Both oaks and pines exhibited significant negative relationships between canopy stomatal conductance and lnVPD, with oaks having a slope term (δG_S_/δ lnVPD) that was significantly more negative (P < 0.001) compared to pines (Figures 6A,B). However, when stomatal sensitivity ratios, which represent stomatal sensitivity to VPD (δG_S_/δ lnVPD) normalized by G_Sref_, were calculated for each individual, oaks had a significantly (*P* = 0.003) lower ratio (0.57 ± 0.02) compared with pines (0.64 ± 0.04). For oaks, stomatal sensitivity ratios were not significantly different (*P* = 0.3) across species and *Q. alba* had the highest ratio (0.63 ±0.04) followed by *Q. velutina* (0.55 ± 0.03) and *Q. prinus* (0.53 ± 0.03). However stomatal sensitivity ratios did differ significantly (*P* < 0.001) for pines across sites, and individuals at the BTB site had the highest ratio (0.88 ± 0.06) followed by individuals at the SL site (0.54 ± 0.07) and individuals at the CB site (0.49 ± 0.02), which were not significantly different (*P* = 0.85) from one another. Pines in all sites exhibited significant positive relationships between canopy stomatal conductance and soil moisture measured in the top 30 cm, while only *Q. velutina* exhibited a positive relationship (*P* < 0.001) for the measured oak species (Figures 6C,D). For pines, individuals at the SL site had the largest slope for the relationship between soil moisture and canopy stomatal conductance, followed by pines at the BTB site and pines at the CB site which had the lowest slope term (Figure 6D). The relationship between stomatal sensitivity to VPD (δG_S_/δ lnVPD) and relative soil moisture content was not significant for any oak species (Figure 7A), however pines at all study sites exhibited significant positive relationships (Figure 7B), with pines at the SL site having the highest slope term, followed by pines at the BTB site and pines at the CB site. In contrast, all oak species exhibited significant negative relationships between stomatal sensitivity to VPD and daily PPFD (Figure 7C) while only pines at the BTB site exhibited a significant negative relationship (*P* = 0.001; Figure 7D). For oak species, *Q. velutina* exhibited a greater negative slope term, followed by *Q. alba* and *Q. prinus* which both exhibited similar slope terms.

## Discussion

In an ecosystem where both water and nutrients are limiting, we found that oaks and pines exhibited differing strategies for resource acquisition and resource-use efficiency. In terms of water use, oaks had higher transpiration rates and stomatal conductances per unit leaf area compared with pines, while pines had greater water use efficiency based on gas exchange data. This confirms our hypothesis and could suggest that the large vessels of oaks are needed to overcome the frictional path length hydraulic resistance of pulling water from deeper groundwater sources. These results differ from the findings of Guehl et al. (1995) who found that *Quercus robur* L. had higher iWUE than *Pinus pinaster* Aiton when derived from either gas exchange or stable isotopes. However, we found that while pines had significantly higher WUE based on instantaneous gas exchange data, iWUE estimated via carbon isotope data did not differ significantly between oaks and pines. In contrast to water usage during the summer, for nutrients, particularly nitrogen, oaks had lower N per unit leaf area than pines as well as lower total leaf areas, but had higher photosynthetic N use efficiencies. Based on leaf lifespans, we expected pines to have greater PNUE as Wright et al. (2004) predict that longer-lived leaves should have lower photosynthetic assimilation rates, lower N_area_ and higher LMA than shorter-lived leaves. We did find that pine needles had higher LMA than oaks, but they also had statistically similar A_max_ and greater N_area_ than oak leaves. Therefore, oaks appear to have greater access to water while pines have greater access to N (or more efficient reallocation of N). This may reflect different rooting strategies with oaks being more deeply rooted to access stable sources of groundwater, while pines may have more shallow roots to access N released from the decomposing litter layer.

Across genera, we found a trade-off between using water and using nutrients efficiently possibly due to the spatial segregation between these two resources with stable water sources found in the deeper groundwater while nutrients are found in the litter layer on the soil surface. On the other hand, within genera, pines but not oaks, exhibited a significant relationship between iWUE and PNUE (Figure 5). Guehl et al. (1995) also found that WUE was strongly affected by N content in pines but not in oaks. This could suggest that evergreen leaf habit and/or large LMA could elicit trade-offs between PNUE and iWUE possibly due to the larger impact of mesophyll conductance in leaves with high LMA (Wright et al., 2004). In terms of access to water, Broeckx et al. (2014) found that short rotation poplar exhibited a trade-off between WUE and PNUE only during conditions of limiting soil moisture. Field et al. (1983) theorize that a tradeoff between NUE and WUE should exist across ecosystems along a moisture gradient with plants in dry ecosystems conserving water through lower stomatal conductance and increased WUE at the expense of NUE and plants in wetter ecosystems maximizing NUE with higher rates of stomatal conductance, and hence, greater water use and lower WUE. This could suggest that co-occurring pines in this study have differing access to water, within and across sites, thereby displaying a tradeoff between WUE and PNUE. Oaks, on the other hand, may only occur in locations where they can access groundwater, and therefore do not exhibit a relationship between WUE and PNUE. However, while oaks exhibited higher PNUE and pines exhibited higher WUE, we found that the relationships between various physiological parameters and these resource-use efficiency estimates were largely similar between the two co-occurring genera. Oaks and pines displayed similar slopes in the linear regression relationships between iWUE_inst._and A_net_ and g_s_ (Figure 3) as well as between PNUE and A_net_, LMA, and N_area_ (Figure 4).

The other main objective of this study was to compare canopy stomatal conductance response to environmental drivers between oaks and pines. Oaks tended to have higher negative slope terms in the relationship between lnVPD and canopy stomatal conductance (δG_S_/δ ln VPD; Figures 6A,B) suggesting greater stomatal sensitivity to VPD. This differs from the findings of Kolb and Stone (2000) who found that stomatal conductance of ponderosa pine (*Pinus ponderosa* Douglas ex C. Lawson) was more sensitive to VPD than Gambel oak (*Quercus gambelii* Nutt.). However, when these slope terms were scaled by reference stomatal conductance at VPD = 1 kPa, (G_Sref_), these stomatal sensitivity ratios were significantly lower in oaks compared with pines. We expected that oaks would exhibit more anisohydric stomatal behavior and therefore exhibit lower stomatal sensitivities compared with pines. However, differences were largely driven by the high stomatal sensitivity ratios measured in pines at the BTB site, and when this site was excluded, stomatal sensitivity ratios are not significantly different (*P* = 0.13) between oaks and pines. A similar stomatal sensitivity between oaks and pines is in accordance with findings of Martínez-Vilalta et al. (2014) who found both oaks and pines to be partially isohydric based on relationships between predawn and midday leaf water potentials. Oren et al. (1999) report an average stomatal sensitivity ratio across tree and ecosystem types of around 0.6, which is consistent with theory and equations of stomatal optimization presented by Katul et al. (2009). Stomatal sensitivity ratios of pines in this study when all sites were included did not differ significantly from this 0.6 value (*P* = 0.06), but were significantly lower (*P* = 0.002) when individuals from the BTB site were excluded. Likewise, oaks from all measured species in this study had stomatal sensitivity ratios that were significantly lower (*P* = 0.04) than the 0.6 value. A stomatal sensitivity ratio lower than 0.6 was also seen in older *Picea mariana* [(Mill.) Britton, Sterns and Poggenburg (Ewers et al., 2005)], desert creosotebush [*Larrea tridentata* (DC.) Coville] (Ogle and Reynolds, 2002) and three oak species (*Q. prinus* L., *Q. alba* L., and *Q. rubra* L.) growing in Central Pennsylvania (Meinzer et al., 2013). For oaks and pines growing in the New Jersey Pinelands, a lower stomatal sensitivity ratio may reflect access to deeper sources of stable water decreasing the sensitivity to atmospheric moisture stress.

Although both oaks and pines in this study appear to have access to a stable source of groundwater, we did find a positive relationship between soil moisture in the upper 30 cm and average canopy stomatal conductance in pines from all sites but only in *Q. velutina* among the measured oak species (Figures 6C,D). Similar results have been reported from other pine-oak systems by Zweifel et al. (2007) and Poyatos et al. (2008), who found that the stomata of Scots pine (*Pinus sylvestris* L.) were more sensitive to drought than pubescent oak (*Q. pubescens* Willd.) and Eilmann et al. (2009) who found that growth in Scots pine was more dependent on water availability than oaks. Likewise, Kolb and Stone (2000) found that oaks in the Southwestern U.S. had higher predawn leaf water potentials than pines suggesting reliable access to deeper water. Both *Q. prinus* and *Q. alba* showed no relationship between canopy stomatal conductance and shallow soil moisture, which suggests that pines have more shallow roots than oaks and are more affected by surface water stress. Differences in source water δ^18^O from oaks and pines at the SL site also indicate differences in rooting depth between pines and oaks and between *Q. prinus* and *Q. velutina* (Song et al., 2014) as suggested by the stomatal conductance data presented in this study. Likewise, pines at all sites exhibited significant positive relationships between stomatal sensitivity to VPD and relative soil water content, while for oak species, none of the relationships were significant (Figures 7A,B). The lower stomatal sensitivity at lower relative soil water content seen in pines is likely the result of decreased reference stomatal conductance rates during drought conditions (Domec et al., 2009). In contrast, all oak species exhibited significant negative relationships between stomatal sensitivity to VPD and daily PPFD, while only pines at the BTB site exhibited a significant negative relationship (Figures 7C,D). Therefore, stomatal responses to VPD were more affected by energy availability than soil moisture in oaks, while the opposite was true for pines.

Our results at the leaf- and individual tree-level can also be compared with ecosystem measurements to better understand how estimates of plant function compare across scales. On a per unit leaf area basis, oaks, and pines had similar maximum photosynthetic assimilation rates while oaks had greater stomatal conductance. On a tree-level basis, pines had greater leaf areas and whole-canopy transpiration rates (E_C_), while oaks had greater leaf-specific transpiration rates (E_L_) and average canopy stomatal conductances. Interestingly, when comparing stomatal conductances between oak and pines, they differed by about 0.1 mol m^−2^ s^−1^ both in leaf-level stomatal conductance estimates and in canopy-level integrated estimates. Across study sites, pines at the BTB site displayed differing physiological responses to environmental drivers (particularly VPD and PPFD) as well as lower iWUE based on carbon isotopes compared with the other sites (SL and CB) that were more similar to one another. This is interesting given that the SL and CB sites are oak-dominated and pine-dominated respectively and the BTB site occurs between the other sites on a latitudinal gradient. Therefore, we can detect no inherent biologic or edaphic features that would explain the differences in pines at the BTB site compared with the other two sites. In terms of stand-level measurements, eddy covariance data from the “SL” oak stand and the “CB” pine stand found that annual gross ecosystem productivity (GEP) was higher in the pine stand, while daily summer GEP, net ecosystem exchange, and ecosystem water use efficiency were higher in the oak stand (Clark et al., 2014). Likewise, we found that iWUE based on carbon isotopes, which is a more temporally integrated measure, was somewhat higher (but not significantly so) in oaks compared with pines even though instantaneous WUE was greater in pines. The different interpretation of WUE at different spatial and temporal scales is likely due to several factors including differences in leaf area and leaf habit with pines assimilating more carbon in the spring and fall which occurs at a lower WUE (Clark et al., 2014) and in an atmosphere with a different carbon isotopic composition than the summer, lowering the iWUE estimate based on carbon isotopes for pines relative to instantaneous, summer values. In terms of total ecosystem water use, daily summer evapotranspiration (ET) rates were similar in oak and pine stands, but annual rates were greater in the pine stand (Clark et al., 2012) likely due to differences in leaf area display whereby evergreen needles increase rainfall interception losses, and therefore, evaporation rates, as well as transpiring in the winter. Therefore, differences in stand basal area, understory function, and the proportion of evaporation to transpiration in ET affect scaling of physiological functioning from the leaf to tree to ecosystem level.

In total, these results can inform predictions of forest function in terms of water use and carbon sequestration given changes in climate and/or changes in species composition as a result of disturbance events that preferentially target oaks or pines. Disturbance is important in many pine-oak ecosystems with both genera being susceptible to wildfire, but each having species with the ability to resprout following fire. Invasive insects can also differentially affect oaks and pines. Gypsy moths and other defoliators tend to preferentially target oaks and other broad-leaved species while many bark beetles specifically target pine species. Several studies have reported predictions of climate change effects on pine-oak ecosystems in Mexico (Gómez-Mendoza and Arriaga, 2007) and the Alps (Weber et al., 2007) suggesting that pines were more vulnerable to drought stress than oaks. Our study also suggests that canopy stomatal conductance in pines is more negatively affected by decreases in precipitation and soil moisture as well as increases in temperature and VPD. In contrast, oaks in our study show greater stomatal sensitivity to changes in energy availability in terms of PPFD and cloud cover, and are less negatively affected by drought stress. Changes in nutrient cycling, particularly in terms of N, should also have a large impact on productivity of forests in the New Jersey Pinelands. For other sandy soil ecosystems, fertilization has been shown to have a larger effect on productivity via increases in leaf area compared to irrigation alone (Albaugh et al., 1998; Ewers et al., 1999) suggesting that trees in these sites are primarily nutrient-limited and may not experience frequent drought stress. In terms of species-specific differences in the New Jersey Pinelands, our study suggests that oaks will be more positively affected by nutrient additions to the ecosystem compared with pines. In terms of increasing atmospheric CO_2_ concentrations, productivity in pines should be more positively affected compared with oaks due to their stomatal sensitivity to soil and atmospheric moisture deficits. In total, these results on resource acquisition and trade-offs between water- and photosynthetic N-use efficiency can inform how oaks and pines function within a given ecosystem, their spatial distribution within the larger landscape, and their potential responses to future environmental change.

## Author contributions

HJR analyzed data and wrote the manuscript, NJC performed data acquisition and manuscript editing, KLC provided data and comments on manuscript, and KVRS provided comments on the manuscript and the data analysis as well as funding, equipment, experimental design.

### Conflict of interest statement

The authors declare that the research was conducted in the absence of any commercial or financial relationships that could be construed as a potential conflict of interest.
