# Supplementary material for: Resource use and efficiency, and stomatal responses to environmental drivers of oak and pine species in an Atlantic Coastal Plain forest
Source: Front Plant Sci. 2015 May 7;6:297. doi: 10.3389/fpls.2015.00297 (PMC4423344; doi:10.3389/fpls.2015.00297)
Supplement: Supplementary file 1 [file Table1.DOCX]

**Table S1:** Means and standard error of gas exchange, leaf isotope and nutrient parameters across *Quercus* individuals in each species measured at the SL site. Rows with different letters differ at α < 0.05.

|  | ***Q. alba*** | ***Q. prinus*** | ***Q. velutina*** |
| --- | --- | --- | --- |
| Maximum assimilation rate (μmol m^-2^ s^-1^) | 14.7 (0.5)**^a^** | 15.5(0.9)**^a^** | 17.9 (0.8)**^b^** |
| Quantum yield (μmol μmol^-1^) | 0.043 (0.004)**^a^** | 0.045 (0.005)**^ab^** | 0.055 (0.003)**^b^** |
| Light compensation pt. (μmol m^-2^ s^-1^) | 23.5 (5.2)**^a^** | 16.8 (4.6)**^a^** | 23.7 (2.7)**^a^** |
| Dark respiration rate (μmol m^-2^ s^-1^) | 0.82 (0.13)**^a^** | 0.80 (0.20)**^a^** | 1.21 (0.15)^a^ |
| V_Cmax,25_ (μmol m^-2^ s^-1^)^1^ | 87.9 (12.5)**^ab^** | 62.8 (5.5)**^a^** | 99.2 (7.3)**^b^** |
| J_max,25_ (μmol m^-2^ s^-1^)^2^ | 79.7 (6.1)**^a^** | 79.3 (8.6)**^a^** | 132.0 (18.5)^b^ |
| TPU_,25_ (μmol m^-2^ s^-1^)^3^ | 5.6 (0.3)**^a^** | 5.5 (0.6)**^a^** | 8.6 (1.1)**^b^** |
| Daytime respiration rate (μmol m^-2^ s^-1^) | 2.9 (0.6)^ab^ | 1.8 (0.4)^a^ | 6.0 (1.4)^b^ |
| Transpiration (E; mmol m^-2^ s^-1^) | 5.02 (0.47)**^ab^** | 4.28 (0.44)**^a^** | 5.89 (0.53)**^b^** |
| Stomatal conductance (g_s_; mol m^-2^ s^-1^) | 0.24 (0.02)**^a^** | 0.21 (0.02)**^a^** | 0.31 (0.03)**^b^** |
| c_i_/c_a_ _inst._^4^ | 0.69 (0.02)**^a^** | 0.68 (0.01)**^a^** | 0.73 (0.02)**^b^** |
| WUE_inst._ (A/E; μmol mmol^-1^)^5^ | 3.25 (0.31)**^a^** | 4.11 (0.27)**^a^** | 3.25 (0.36)**^a^** |
| iWUE_inst._ (A/g_s_; μmol mol^-1^)^6^ | 64.1 (3.8)**^b^** | 70.2 (3.4)**^a^** | 57.6 (3.6)**^a^** |
| Ball-Berry parameter (m) | 9.9 (1.6)**^a^** | 7.0 (1.1)**^ab^** | 6.6 (1.0)**^b^** |
| δ^13^C (‰)^7^ | -30.1 (0.2)**^a^** | -29.5 (0.4)**^a^** | -29.5 (0.2)**^a^** |
| Δ (‰)^8^ | 19.4 (0.3)**^a^** | 19.0 (0.5)**^a^** | 18.9 (0.2)**^a^** |
| c_i_/c_a_ _iso._^9^ | 0.66 (0.01)**^a^** | 0.64 (0.02)**^a^** | 0.64 (0.008)**^a^** |
| iWUE_iso._ (μmol mol^-1^)^10^ | 84.6 (3.6)**^a^** | 89.9 (5.4)**^a^** | 89.9 (2.0)**^a^** |
| Leaf mass per unit area (LMA; g m^-2^) | 77.9 (1.6)**^a^** | 106.9 (5.3)**^b^** | 113.7 (3.2)**^b^** |
| Leaf nitrogen concentration (N; %) | 2.31 (0.04)**^a^** | 2.03 (0.04)**^b^** | 2.14 (0.07)**^b^** |
| Leaf carbon concentration (C; %) | 48.7 (0.1)**^a^** | 48.2 (0.6)**^a^** | 48.1 (1.1)**^a^** |
| Leaf C/N ratio | 21.3 (0.4)**^a^** | 23.9 (0.4)**^b^** | 22.7 (0.3)**^b^** |
| Nitrogen per unit leaf area (N_area_; g m^-2^) | 1.80 (0.04)**^a^** | 2.17 (0.11)**^b^** | 2.47 (0.08)**^b^** |
| PNUE (μmol g^-1^ s^-1^)^11^ | 7.69 (0.15)**^a^** | 6.77 (0.40)**^a^** | 6.77 (0.32)**^a^** |

^1^Rubisco-limited carboxylation rate at 25 °C, ^2^Electron transport-limited carboxylation rate at 25 °C, ^3^Triose phosphate utilization-limited carboxylation rate at 25 °C, ^4^Instantaneous ratio of [CO_2_]_inside leaf_ to [CO_2_]_ambient air_, ^5^Instantenous water-use efficiency, ^6^Instantenous intrinsic water–use efficiency, ^7^Leaf isotopic ratio, ^8^Leaf isotopic discrimination, ^9^Ratio of [CO_2_]_inside leaf_ to [CO_2_]_ambient air_ based on carbon isotope discrimination, ^10^Intrinsic water-use efficiency based on carbon isotope discrimination, ^11^Photosynthetic nitrogen-use efficiency
