# Supplementary material for: Resource use and efficiency, and stomatal responses to environmental drivers of oak and pine species in an Atlantic Coastal Plain forest
Source: Front Plant Sci. 2015 May 7;6:297. doi: 10.3389/fpls.2015.00297 (PMC4423344; doi:10.3389/fpls.2015.00297)
Supplement: Supplementary file 2 [file Table2.DOCX]

**Table S2:** Means and standard error of gas exchange, leaf isotope and nutrient parameters across *Pinus* individuals (*P. rigida* and *P. echinata*) measured at the SL (oak-dominated stand), BTB and CB (pine-dominated stands) sites. Rows with different letters differ at α < 0.05.

| ***Pinus spp.*** | SL | BTB | CB |
| --- | --- | --- | --- |
| Maximum assimilation rate (μmol m^-2^ s^-1^) | 15.3 (1.5)**^a^** | 15.5 (1.5)**^a^** | 18.4 (1.0)**^a^** |
| Quantum yield (μmol μmol^-1^) | 0.047 (0.007)**^a^** | 0.047 (0.004)**^a^** | 0.048 (0.004)**^a^** |
| Light compensation pt. (μmol m^-2^ s^-1^) | 18.6 (1.8)**^a^** | 30.9 (6.4)**^a^** | 14.4 (2.6)**^a^** |
| Dark respiration rate (μmol m^-2^ s^-1^) | 0.79 (0.15)**^a^** | 1.30 (0.27)**^a^** | 0.67 (0.13)^a^ |
| V_Cmax,25_ (μmol m^-2^ s^-1^)^1^ | 84.3 (5.0)**^a^** | 82.0 (12.9)**^a^** | 82.9 (7.5)**^a^** |
| J_max,25_ (μmol m^-2^ s^-1^)^2^ | 114.4 (9.1)**^a^** | 76.3 (12.4)**^a^** | 94.5 (11.6)**^a^** |
| TPU_,25_ (μmol m^-2^ s^-1^)^3^ | 8.4 (0.4)**^a^** | 5.1 (0.7)**^a^** | 7.2 (1.0)**^a^** |
| Daytime respiration rate (μmol m^-2^ s^-1^) | 6.7 (0.9)^a^ | 3.0 (0.6)^b^ | 3.6 (0.5)^b^ |
| Transpiration (E; mmol m^-2^ s^-1^) | 3.70 (0.47)**^a^** | 5.32 (0.36)**^b^** | 5.08 (0.34)**^b^** |
| Stomatal cond. (g_s_; mol m^-2^ s^-1^) | 0.14 (0.02)**^a^** | 0.18 (0.02)**^a^** | 0.21 (0.01)**^a^** |
| c_i_/c_a_ _inst._^4^ | 0.55 (0.04)**^a^** | 0.65 (0.02)**^b^** | 0.65 (0.01)**^b^** |
| WUE_inst._ (A/E; μmol mmol^-1^)^5^ | 4.34 (0.71)**^a^** | 2.87 (0.24)**^b^** | 3.55 (0.35)**^ab^** |
| iWUE_inst._ (A/g_s_; μmol mol^-1^)^6^ | 105.0 (11.3)**^a^** | 85.1 (4.3)**^a^** | 87.1 (5.8)**^a^** |
| Ball-Berry parameter (m) | 3.7 (1.1)**^a^** | 5.1 (1.2)**^a^** | 7.5 (3.5)**^a^** |
| δ^13^C (‰)^7^ | -30.0 (0.3)**^a^** | -30.8 (0.2)**^b^** | -30.1 (0.3)**^a^** |
| Δ (‰)^8^ | 19.6 (0.3)**^a^** | 21.4 (0.4)**^b^** | 19.8 (0.4)**^a^** |
| c_i_/c_a_ _iso._^9^ | 0.67 (0.01)**^a^** | 0.75 (0.02)**^b^** | 0.68 (0.02)**^a^** |
| iWUE_iso._ (μmol mol^-1^)^10^ | 81.5 (3.0)**^a^** | 61.8 (4.6)**^b^** | 79.1 (4.3)**^a^** |
| Leaf mass per unit area (LMA; g m^-2^) | 229.2 (18.4)**^a^** | 241.7 (8.4)**^a^** | 251.2 (9.5)**^a^** |
| Leaf nitrogen concentration (N; %) | 1.16 (0.03)**^a^** | 1.01 (0.03)**^b^** | 0.92 (0.01)**^b^** |
| Leaf carbon concentration (C; %) | 47.8 (0.3)**^a^** | 49.5 (0.8)**^a^** | 47.2 (0.3)**^a^** |
| Leaf C/N ratio | 41.5 (1.1)**^a^** | 49.8 (1.3)**^b^** | 51.5 (0.8)**^b^** |
| Nitrogen per unit leaf area (N_area_; g m^-2^) | 2.67 (0.25)**^a^** | 2.44 (0.09)**^a^** | 2.30 (0.09)**^a^** |
| PNUE (μmol g^-1^ s^-1^)^11^ | 5.29 (0.68)**^a^** | 5.77 (0.74)**^a^** | 7.44 (0.40)**^a^** |

^1^Rubisco-limited carboxylation rate at 25 °C, ^2^Electron transport-limited carboxylation rate at 25 °C, ^3^Triose phosphate utilization-limited carboxylation rate at 25 °C, ^4^Instantaneous ratio of [CO_2_]_inside leaf_ to [CO_2_]_ambient air_, ^5^Instantenous water-use efficiency, ^6^Instantenous intrinsic water–use efficiency, ^7^Leaf isotopic ratio, ^8^Leaf isotopic discrimination, ^9^Ratio of [CO_2_]_inside leaf_ to [CO_2_]_ambient air_ based on carbon isotope discrimination, ^10^Intrinsic water-use efficiency based on carbon isotope discrimination, ^11^Photosynthetic nitrogen-use efficiency
